# Comparative Proteomic Profiling of *Leishmania tropica*: Investigation of a Case Infected with Simultaneous Cutaneous and Viscerotropic Leishmaniasis by 2-Dimentional Electrophoresis and Mass Spectrometry

**Published:** 2015

**Authors:** Homa HAJJARAN, Parisa MOUSAVI, Richard BURCHMORE, Mehdi MOHEBALI, Mitra MOHAMMADI BAZARGANI, Ghasem HOSSEINI SALEKDEH, Elham KAZEMI-RAD, Mohammad Reza KHORAMIZADEH

**Affiliations:** 1 *Dept. of Medical Parasitology and Mycology, School of Public Health, Tehran University of Medical Sciences, Tehran, Iran*; 2 *Institute of Infection, Immunity and Inflammation, College of Medical, Veterinary and Life Science, University of Glasgow, United Kingdom*; 3 * Research Center for Endemic Parasites of Iran, Tehran University of Medical Sciences, Tehran, Iran*; 4 *Agriculture institute, Iranian Research organization for Science and Technology, Tehran, Iran*; 5 *Agricultural Biotechnology Research Institute of Iran, (ABRI), Karaj, Iran*; 6 *Royan Institute for Stem Cell Biology and Technology, ACCER, Tehran, Iran*; 7 *Dept. of Medical Parasitology, Pasteur Institute of Iran, Tehran, Iran*; 8 *Biosensor Research Center, Endocrinology and Metabolism Molecular-Cellular Sciences Institute, Tehran University of Medical Sciences, Tehran, Iran *; 9 *Dept. of Medical Biotechnology, School of Advanced Technologies in Medicine, Tehran University of Medical Sciences, Iran*

**Keywords:** *L. tropica*, Viscerotropic leishmaniasis, Proteome, 2-DE, LC mass spectrometry

## Abstract

***Background:*** Viscerotropic leishmaniasis caused by *Leishmania tropica* poses a significant problem in the diagnosis and treatment management. Since differential gene expression is more important in outcome of the infection, we employed proteomic approach to identify potential proteins involved in visceralization of *L. tropica*.

***Methods:*** The proteomes profiling of *L. tropica *isolated from cutaneous and visceral tissues of one host were compared by 2-DE/MS proteomics study. Moreover, the transcript level of some identified proteins was confirmed using real-time RT-PCR.

***Results:*** Of the 700 protein spots that were detected reproducibly on each gel, 135 were found to be differentially expressed (*P*≤ 0.05). Most of responsive proteins in visceral isolate changed in less abundant compared to cutaneous isolate. Among differentially expressed proteins, 56 proteins were confidently identified and classified according to the biological process. The largest groups consist of proteins involved in carbohydrate metabolism and protein synthesis. Most of the identified proteins, which implicated in energy metabolism, cell signaling and virulence were down-regulated, whereas some proteins that have a role in protein folding, antioxidant defense and proteolysis were up-regulated in visceral form. Moreover, the transcript level of some identified proteins such as co-chaperon was confirmed using real-time RT-PCR.

***Conclusion:*** *L. tropica* probably uses different mechanisms for survival and multiplication in viscera to establish viscerotropic leishmaniasis. The current study provides some clues into the mechanisms underlying the dissemination of *L. tropica.*

## Introduction

The order Kinetoplastidae includes a large number of pathogenic parasite species, which could infect a wide range of hosts, including humans, canine, rodents etc. There are approximately 21 species of the genus *Leishmania* cause leishmaniasis. Depending on the involved species, infection could consist of a spectrum of disease ranging from simple self-limiting cutaneous forms, namely cutaneous leishmaniasis (CL), mucocutaneous leishmaniasis (MCL) to a rather fatal if untreated, visceral leishmaniasis (VL) (1, 2). These clinical forms are primarily attributed to the species of parasites involved and the host immune system, although the mechanisms of species tissue tropism are largely unknown (3). 

However, the species of *Leishmania* is the main factor that determines the clinical presentation. For example, in the old world and south Asia including Iran, *L. tropica* and *L. major* are the causative agents of CL and *L. infantum *is responsible for VL (4, 5). In recent decades, exceptional cases have been observed, such as visceral outcomes in infected individuals with *L. tropica* and cutaneous outcomes in cases infected with *L. infantum *(6- 8). Moreover, other confirmed cases were reported in human patients and animal reservoirs such as canine (dogs) which referred to as viscerotropic leishmaniasis (VTL) (9, 4). In fact, viscerotropic leishmaniasis is a concomitant form of cutaneous and visceral disease caused by *L. tropica*. Unlike classical VL, the variable pathology, lacking hepato splenomegaly and lower serum titers of anti-leishmania antibodies were observed in infected VTL cases (9). 

The availability of the genome sequences will serve in ongoing efforts to study the parallel expression of genes and protein contents by variety of proteomic approaches like 2-dimentional gel electrophoresis (2-DE) and mass- spectrometry (10,11). In this regard, proteomics will permit the determination of how parasites interact with their hosts, respond to anti parasitic drugs and develop mechanisms to escape from immune response (12). 

In this study, we employed proteomic approach for the first time in order to identify potential proteins implicated in disseminating of *L. tropica* from cutaneous to the viscera. Two cutaneous and visceral *L. tropica* isolates were subjected to 2-DE analysis by using pH [4-7] followed by liquid chromatography mass spectrometry (LC/MS) for protein identification. Here the goal was to identify differential protein abundance that may possible represent in the process of viscerotropism in *L. tropica*.

## Materials and Methods

***Leishmania tropica isolates***

We used the *Leishmania *isolates obtained from a 5-month old owner dog with multiple cutaneous lesions from Karaj, central Iran was referred to Faculty of Veterinary Medicine and School of Public Health, Tehran university of Medical Sciences (13). The animal had no systemic clinical signs. Biopsy specimens were collected aseptically from cutaneous and visceral tissues including spleen and liver (was later named cutaneous isolate (CI) and viscerotropic isolate (VTI)). 

Parasites were grown in RPMI 1640 (Gibco, Germany) with 20% FBS (Gibco) at 23 ºC. The identities of the isolates were obtained by DNA extraction from all the obtained tissues, skin, spleen, and liver followed by PCR amplification of NAGT gene and RFLP and sequencing (14). The nucleotide sequence data were submitted to the GenBank database and registered with accession numbers HM234011 and HM234012 for visceral and cutaneous tissues, respectively. 

***Protein extraction***

Proteomics analysis was performed on triplicate cultured promastigotes obtained from both cutaneous and visceral tissues. Promastigotes were harvested by centrifugation at 3000 rpm for 20 min at 4 ºC and washed in sterile PBS, pH: 7.2-7.4 for 10 min three times. The cells were resuspended in 5 mM Tris–HCl, pH 7.8, containing 1 mM PMSF (Merck, Germany) as a protease inhibitor. The samples were sonicated at 40 Hz 3 times for 10 s with 50 s intervals on ice bath. Proteins were precipitated by 10% (w/v) TCA (Merck, Germany) in acetone (Merck) with 0.07 % (w/v) DTT (Merck) for 1 hour at -20 °C. The samples were then centrifuged at 17500 g (Hettich, Germany) for 15 minutes at 4 °C the pellets were washed with ice-cold acetone containing 0.07% DTT, incubated at -20 °C for 1 h and centrifuged at 4 °C. Washing and sedimentation of the pellets repeated three times and then residual acetone was allowed to air-dry. The samples powders were then solubilized in lysis buffer [9.5 M urea (Merck), 2% (w/v) CHAPS (Merck), 0.8% (w/v) Ampholyte (Bio-Rad, USA) pH 3-10, 1% (w/v) DTT]. The concentration of protein was measured by the Bradford assay (Bio-Rad, USA) with BSA (Merck) as the standard.

***2-Dimentional Electrophoresis***

The isoelectric focusing and the second dimension were performed as previously described with some modifications (15). For analytical and preparative gels, 120 μg and 1.2 mg of extracted promastigotes proteins were loaded respectively. Isoelectric focusing was carried out on the 18 cm immobilized pH gradient (IPG) strips (pH 4-7) (Bio-Rad, USA). IPG strips were rehydrated overnight by loading the samples diluted with rehydration buffer containing 8 M urea, 4% CHAPS, 2% ampholyte, 50 mM DTT, and traces of bromophenol blue (Merck). Isoelectric focusing (IEF) was conducted at 20 °C with Mutiphor II and a DryStrip kit (GE Healthcare, Germany). The running condition was as follows: 300 V for 90 minute, followed by 500 V for 90 min, 1000 V for 3 hour and finally 3500 V for 16 h. The focused strips were equilibrated twice for 15 min in 10 ml equilibration solution. The first equilibration was performed in a solution containing 6 M urea, 20% (w/v) glycerol, 2% (w/v) SDS (Merck), 1% (w/v) DTT, and 50 mM Tris-HCl (Merck) buffer, pH 8.8. The second equilibration was performed in a solution with 2.5% (w/v) iodoacetamide (Merck). Separation in the second dimension was performed by SDS-PAGE in a vertical slab of acrylamide (Merck) (12% total monomer, with 2.6% cross-linker) using a PROTEAN II Multi Cell (BioRad). The protein spots in analytical and preparative gels were visualized by silver nitrate (Merck, Germany) and CBB/ G-250 (Sigma, Germany) respectively (16, 17). 

***Staining and gel image analysis***

GS-800 densitometer (Bio-Rad) at a resolution of 600 dots per square inch (dpi) was used for scanning of silver stain gels. The scanned gels saved as TIF images for subsequent analysis. Gels were analyzed using the Melanie 6 software (GeneBio, Geneva, Switzerland). Spot detection, protein quantification, and spot pairing were carried out based on software settings. The molecular masses of protein on gels were determined by co electrophoresis of standard protein markers (GE Heathcare) and* pI* of the proteins were determined by migration of the protein spots on 18 cm IPG (pH 4-7, linear) strips. The percent volume of each spot was estimated and analyzed to protein abundance determination. 2-dimensional gel per sample was run for three biologically independent replicates. Spots were determined to be significantly up- or down-regulated when *P*< 0.05. The induction factor (IF) was calculated by dividing the percent volume of spots in viscerotropic isolate (VTI) to the percent volume of spots in cutaneous isolate (CI). Statistical analysis of protein variations was carried out using the Student *t*-test with a confidence level of 95% on relative volume of matched spots.

***Protein digestion, peptide extraction and mass analysis***

The proteins spots that showed significant statistically changes in VTI compare to CI were excised from the CBB, stained 2-DE gels and subjected to in-gel trypsin digest as described previously (18). Peptides were solubilized in 0.5% formic acid and fractionated on a nanoflow uHPLC system (Thermo RSLCnano) before online analysis by electrospray ionisation (ESI) MS on an Amazon ion trap MS/MS (Bruker Daltonics). Peptide separation was performed on a Pepmap C18 reversed phase column (LC Packings), using a 5 - 85% v/v acetonitrile gradient (in 0.5% v/v formic acid) run over 45 min. at a flow rate of 0.2 l / min. Mass spectrometric (MS) analysis was performed using a continuous duty cycle of survey MS scan followed by up to ten MS/MS analyses of the most abundant peptides, choosing the most intense multiply charged ions with dynamic exclusion for 120 s.MS data was processed using Data Analysis software (Bruker) and the automated Matrix Science Mascot Daemon server (v2.1.06). Protein identifications were assigned using the Mascot search engine to interrogate in house databases of protein sequences for *L. major*. In all identiﬁed proteins, the probability score was greater than the one ﬁxed by MASCOT as being signiﬁcant, that is, a *p* value < 0.05.

***Real-Time RT-PCR analysis***

***RNA extraction and cDNA synthesis***

Total RNA was extracted from 10^8^ promastigotes of cutaneous and viscerotropic *L. tropica* isolates during the early stationary phase using Tripure reagent (Roch, Mannheim, Germany) according to the manufacture’s instruction. The quantity and quality of RNA were analyzed using nanodrop (ND-1000, Thermo Scientific Fisher, US) and gel electrophoresis, respectively. The RNAs were treated with RNase-free DNase I (Fermentas, Burlington, Canada) to avoid any genomic contamination. Complementary DNA (cDNA) was synthesized from 1 µg of total RNA using Transcriptor first strand cDNA synthesis Kit (Roch, Mannheim, Germany) following the manufacturer’s instructions. 

***Real-time RT-PCR analysis***

Real-time reverse transcriptase-PCR (RT-PCR) was conducted to investigate the differences in gene expression of a number of dominant proteins between cutaneous and viscerotropic *L. tropica* isolates. Target gene primers were designed by primer 3 software version 0.4.0 (http://frodo.wi.mit.edu/) according to the identified proteins (Table 1). Glyceraldehyde 3-phosphate dehydrogenase (GAPDH) gene was included for normalization purposes, referred as internal control. RT-PCR was performed in 20 µl reactions containing 1 µl cDNA target, 100 nM forward and reverse primers and 1x SYBR^® ^Premix Ex Taq ^TM ^II (Takara, Tokyo, Japan). Experiments were carried out in triplicate using a StepOne ^TM^ Real-Time PCR System (Applied Biosystems, Life Technologies, USA). The PCR condition was as follows: activation at 95 °C for 30 s, amplification at 95 °C for 5 s, 60 °C for 30 s for 40 cycles. The relative value of the expression level of each gene was determined based on the threshold cycle (CT) value of the target genes, normalized to that of reference genes (GAPDH) using the 2^-∆∆ct ^method and the level of significance acceptable was 95% (*P*<0.05) (19).

## Results

***Proteomic response***

**Fig. 1:**
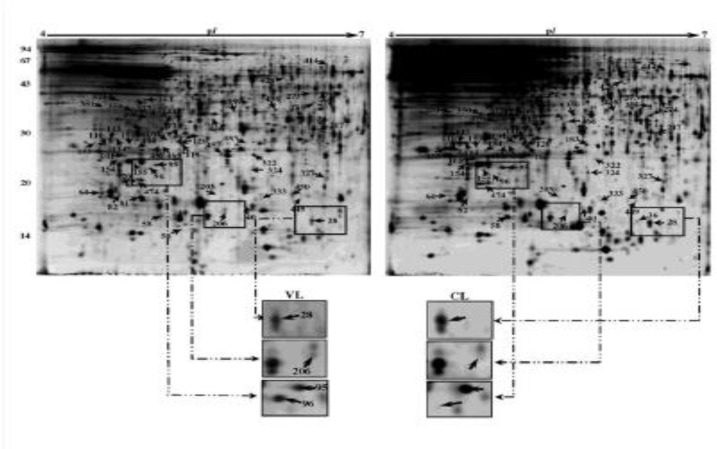
2-D gel analysis of proteins extracted from *Leishmania tropica* isolated from cutaneous and visceral (spleen) of a 5-month old dog. In first dimension (IEF), 120 µg was loaded on a 18-cm IPG strip with a linear gradient of pH 4-7. In the second dimension, 12% SDS-PAGE gels were used, with a well for molecular weight standards. Proteins were visualized by silver staining. Arrows represent spots identified by MS (Tables 2-3). Examples of changes in protein abundance between viscerotropic **(VTI)** and cutaneous **(CI)** samples have been presented

**Fig. 2:**
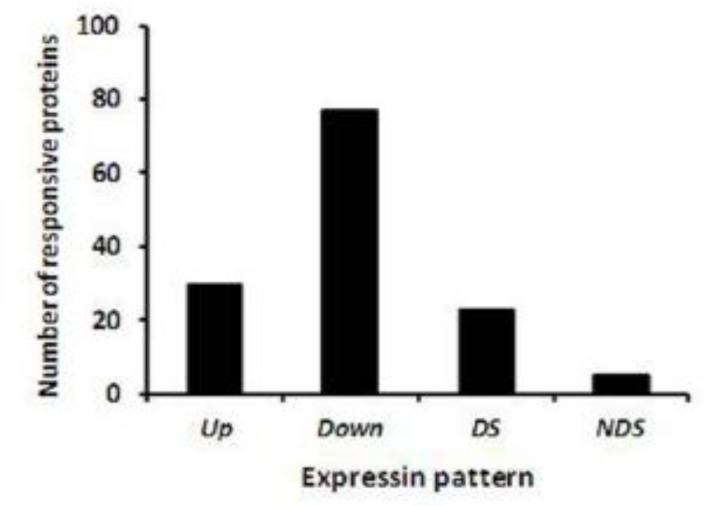
Number of proteins differing significantly in abundance in *Leishmania tropica* isolated from visceral (spleen) (VTI) to cutaneous lesions (CI) of a dog infected to co-cutaneous/viscerotropic leishmaniasis compared.

We analyzed the gels from three independent replicates of both cutaneous and visceral *L. tropica* isolates (6 gels in total). Of the 723 protein spots that were detected reproducibly on each gel, 135 spots showed statistically significant changes (*P*≤ 0.05) in VTI compared to CI (Fig. 1 and Table 2). Most of responsive proteins (107 proteins) in VI changed in abundance compared to CI, and only 28 proteins changed in gel position (present/absent) (Table 2). The majority of changes in VI proteins were seen as decreased in abundance; out of 135 proteins, only 30 proteins increased in abundance, while 77 proteins down-regulated in VI compared to CI. Some 23 proteins were present only in viscera isolate, while five proteins were only observed in CI and absent in VI (Fig. 2 and Table 2). 

**Table 1:** Sequences of Primers used in Real-Time RT-PCR

**Primers name**	**Sequence (5' → 3')**	**Product size**
Triosephosphate isomerase (TPI)	F acacaacatctcccatgacg R gatcggcattgacacttcac	157
Calmodulin-like protein (CLP)	F gctcgacgtggaacctctt R cagcttaatgaatgcgtcgt	164
Co-chaperone GrpE (C**o**-CHP)	F aggcgttttctgccttttc R tggggtcgaactttgtaccta	159
Elongation factor 1-alpha (EF-1 alpha)	F gatcgagaagttcgagaaggag R acttccacagcgcaatgtc	125
Small ubiquitin protein (SUP)	F gatatcgctgaaggtcgtcaa R ccctgcttcttgcagtacg	105
Glyceraldehydes-3-phosphate dehydrogenase (GAPDH)	F gaagtacacggtggaggctg R cgctgatcacgaccttcttc	206

F: Forward, R: Reverse

**Table 2:** Proteins identified using LC/MS analysis

						**Expression pattern. ** f				
						***Cutaneous isolate (CI)***		***Viscerotropic isolate (VTI)***		
**Spot** **No. ** a	**Protein name**	**Accession** **No.** b	**(PI/MW)** **Exp. ** c	**(PI/MW)** **Theo.** d	**Protein Score.** e	**Ave %vol**	**Sd**	**Ave %vol**	**Sd**	**Induction factor (V/C). ** g
*28*	*hypothetical protein*	Q4QFW1	6.38/16	6.1/15	162	0.224	0.019	0.114	0.032	0.51
*36*	*hypothetical protein*	Q4QFW1	6.3/16	6.1/15	79	0.037	0.007	0.000	0.000	*NDS*
*44*	*ADF/Cofilin*	E9ADQ2	5.77/15	5.6/16	219	0.526	0.097	0.109	0.189	0.21
*50*	*60S ribosomal protein L30*	E9AEK1	5.29/15	9.7/11	43	0.000	0.000	0.027	0.009	*DS*
*58*	*hypothetical protein, conserved (Alba superfamily)* *	Q4QGA9	5.12/16	5.2/13	409	0.238	0.014	0.089	0.043	0.37
*64*	*60S ribosomal protein L23a, putative*	Q4QJ20	6.64/19	10.5/16	172	0.039	0.010	0.117	0.017	3.02
*70*	*hypothetical protein, conserved (Sfi1 spindle body protein)* *	Q4Q6E8	4.86/20	10.6/121	72	0.000	0.000	0.107	0.021	*DS*
*81*	*elongation factor 1-alpha*	Q4QEI9	4.96/18	9.0/49	167	0.000	0.000	0.222	0.031	*DS*
*82*	*40S ribosomal protein S12, putative*	Q4QG97	4.79/19	4.8/16	106	0.038	0.010	0.079	0.016	2.08
*95*	*p21 antigen protein*	Q9U8C2	5.1/24	5.2/21	108	0.171	0.006	0.061	0.006	0.36
*96*	*hypothetical protein, unknown function*	E9ACC9	5.04/23	5.3/18	145	0.007	0.002	0.057	0.006	8.38
*101*	*calmodulin-like protein*	Q4QF91	4.8/25	4.9/21	264	0.090	0.008	0.033	0.005	0.37
*110*	*peroxidoxin*	Q4QBH2	4.67/27	6.4/25	205	0.084	0.010	0.031	0.003	0.37
*113*	*proteasome beta 6 subunit, putative*	Q4QJ65	4.71/29	6.5/28	174	0.163	0.037	0.068	0.007	0.42
*114*	*triosephosphate isomerase*	Q4QAP8	4.77/29	8.6/27	216	0.113	0.009	0.038	0.008	0.34
*119*	*vacuolar sorting-like protein*	Q4Q5H7	5.3/28	5.1/22	88	0.075	0.014	0.040	0.002	0.54
*120*	*hs1vu complex proteolytic subunit-like*	Q4Q116	5.28/28	5.7/23	227	0.017	0.005	0.035	0.003	2.03
*131*	*hypothetical protein, conserved*	Q4Q079	5.25/30	5.5/29	61	0.079	0.007	0.038	0.010	0.47
*137*	*hypothetical protein, conserved (SH3 domain protein)* *	Q4QJ54	5.38/32	5.3/31	372	0.014	0.002	0.000	0.000	*NDS*
*144*	*glycosomal malate dehydrogenase*	Q4QDF0	5.1/33	9.0/34	261	0.031	0.001	0.013	0.003	0.42
*151*	*hypothetical protein, conserved*	Q4QED8	4.87/28	5.2/24	196	0.019	0.004	0.004	0.001	0.19
*153*	*peroxidoxin*	Q4QBH2	4.58/28	4.6/25	228	0.102	0.016	0.036	0.013	0.35
*154*	*Qc-SNARE protein, putative*	Q4QIG6	5.78/24	8.5/24	157	0.110	0.011	0.019	0.010	0.17
*155*	*eukaryotic translation initiation factor 1A, putative*	Q4QF06	4.92/23	4.7/19	426	0.043	0.008	0.013	0.010	0.30
*161*	*hypothetical protein, conserved (Alba superfamily)* *	Q4Q2T7	5.2/29	9.8/23	224	0.024	0.001	0.004	0.001	0.18
*183*	*hypothetical protein, conserved (Alba superfamily)* *	Q4Q2T7	5.83/28	9.8/23	176	0.061	0.018	0.012	0.003	0.20
Spot No. a	Protein name	Accession No. b	(PI/MW) Exp. c	(PI/MW) Theo. d	Protein Score. e	Ave %vol	Sd	Ave %vol	Sd	Induction factor (V/C). g
*203*	*alpha tubulin*	Q4QGC5	5.61/19	4.9/50	202	0.016	0.002	0.006	0.001	0.39
*206*	*Tryparedoxin*	E9ADX3	5.64/16	6.6/17	40	0.018	0.001	0.037	0.007	2.11
*217*	*proteasome alpha 1 subunit, putative*	E9AFW0	6.44/31	6.8/27	202	0.058	0.008	0.030	0.006	0.52
*255*	*cytosolic malate dehydrogenase*	Q4Q7X6	6.4/37	5.8/34	493	0.061	0.011	0.032	0.013	0.53
*273*	*NADP-dependent alcohol dehydrogenase, putative*	Q4QBD8	6.37/40	5.8/38	249	0.095	0.004	0.031	0.004	0.33
*274*	*NADP-dependent alcohol dehydrogenase, putative*	Q4QBD8	6.48/40	5.8/38	366	0.287	0.031	0.146	0.019	0.51
*322*	*co-chaperone GrpE, putative*	Q4Q7N4	5.89/26	7.7/24	242	0.019	0.002	0.083	0.011	4.28
*324*	*ADP-ribosylation factor-like protein*	Q4Q756	5.86/23	6.0/20	169	0.053	0.015	0.017	0.001	0.32
*327*	*hypothetical protein, unknown function*	Q4QEA4	6.5/21	9.3/17	46	0.000	0.000	0.027	0.007	*DS*
*333*	*endoribonuclease L-PSP (pb5), putative*	Q4QBF5	5.96/18	5.5/17	228	0.007	0.003	0.016	0.004	2.38
*350*	*heat shock protein-like protein, putative*	Q4Q584	4.9/35	5.0/36	442	0.039	0.008	0.020	0.007	0.52
*353*	*adenylate kinase, putative*	Q4QC71	4.5/39	5.7/30	198	0.069	0.017	0.022	0.004	0.31
*359*	hypothetical protein, conserved (P-loop_NTPase super family)*	Q4QFN8	5.72/36	5.6/32	85	0.074	0.013	0.030	0.012	0.40
*374*	*succinyl-CoA synthetase alpha subunit, putative*	Q4Q9M4	4.77/38	9.2/31	313	0.000	0.000	0.023	0.010	*DS*
*377*	*dihydroorotate dehydrogenase*	Q4QEW7	4.93/39	5.7/35	83	0.030	0.007	0.006	0.003	0.21
*381*	*hypothetical protein, conserved (Enkurin superfamily)* *	Q4QAX0	4.65/36	9.0/31	152	0.015	0.004	0.001	0.000	0.04
*389*	*phosphoglycerate kinase B, cytosolic*	Q4QD33	4.8/42	8.0/45	425	0.000	0.000	0.002	0.000	*DS*
*414*	*Structure-specific endonuclease subunit SLX1 homolog*	Q4Q9W0	6.52/57	8.7/75	29	0.061	0.012	0.029	0.007	0.48
*428*	*elongation factor 1-alpha*	Q4QEI9	6.14/47	9.0/49	202	0.058	0.005	0.009	0.002	0.16
*436*	*succinyl-CoA ligase [GDP-forming] beta-chain, putative*	Q4Q1C4	5.56/33	6.5/44	345	0.000	0.000	0.047	0.012	*DS*
*438*	*hypothetical protein, conserved*	O97202	4.1/36	9.6/46	95	0.015	0.001	0.060	0.012	3.97
*449*	*hypothetical protein, conserved*	Q4QHN3	6.28/18	5.9/16	194	0.017	0.004	0.007	0.002	0.42
*450*	*hypothetical protein, conserved*	Q4QHN3	6.22/19	5.9/16	152	0.015	0.005	0.103	0.025	6.75
*457*	*peroxidoxin*	Q4QBH2	5.62/26	4.6/25	205	0.000	0.000	0.039	0.000	*DS*
*466*	*glycosomal malate dehydrogenase*	Q4QDF0	5.66/33	9.0/34	217	0.024	0.004	0.000	0.000	*NDS*
Spot No. a	Protein name	Accession No. b	(PI/MW) Exp. c	(PI/MW) Theo. d	Protein Score. e	Ave %vol	Sd	Ave %vol	Sd	Induction factor (V/C). g
*468*	*heat shock protein 90*	E9ADS8	5.1/28	5.0/87	182	0.014	0.002	0.006	0.001	0.40
*469*	*hs1vu complex proteolytic subunit-like*	Q4Q116	5.13/27	5.7/23	194	0.018	0.002	0.008	0.002	0.42
*474*	*small ubiquitin protein, putative*	Q4QIC2	4.94/20	5.0/13	126	0.009	0.002	0.019	0.004	2.16
*484*	*peroxidoxin*	Q4QBH2	4.94/27	4.6/25	226	0.044	0.007	0.022	0.008	0.50
*496*	*hypothetical protein, conserved (SNF-7-like protein)* *	Q4Q130	5.02/34	4.8/25	234	0.034	0.005	0.006	0.003	0.18

a The numbering corresponds to the 2-DE gel in Fig. 1./

b Accession number in Swiss-Prot. /

c Experimental *pI* and molecular weight.

d Theoretical *pI* and molecular weight./

e Mascot score greater than 26 were significant at *p*=0.05./

F Expression pattern of spots that showed a statistically change in cutaneous and visceral leishmaniasis./

g The induction factor calculated by dividing the percent volume of spots in gels corresponding to visceral leishmaniasis to the percent volume of spots in cutaneous leishmaniasis samples./ Average percent volume (Ave %vol) and standard deviation (sd) of spots from two samples (cutaneous and visceral) and Three replication have been presented. Spots were concluded to be significantly up- or down-regulated when *p* was <0.05./

* Hypothetical proteins with special domains /The data-base search was run against the NCBI non-redundant protein data-base and Uniprot's Swiss-Prot.

**Up**: proteins more abundant in VTI.

**Down**: proteins less abundant in VTI.

**DS**: detected spots only in VTI (in fact not detected spots in CI(

**NDS**: not detected spots in VTI (in fact detected spots only in CI)

***Protein identification***

**Fig. 3:**
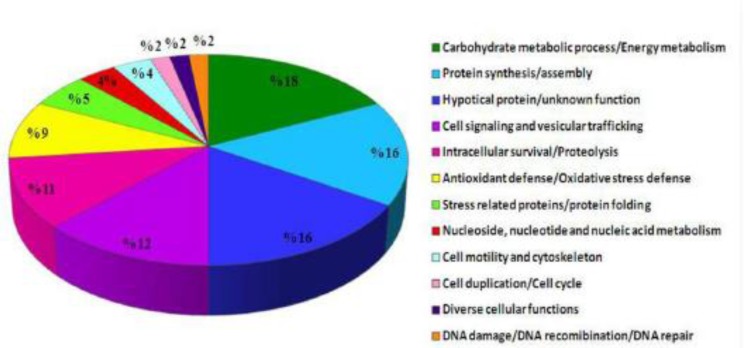
Functional annotation of the identified proteins classified by biological function and processes described in Table 3.

Of 135 differentially expressed proteins detected on the analytical gels, 61 proteins were detected reliably on CBB-stained preparative gels; analyzed using LC/MS/MS after excitation from CBB stained gels. Of these, 56 proteins were identified (Table 2 and Fig. 1). These proteins according to their functions and biological processes were classified in twelve categories: carbohydrate metabolism process, protein synthesis and assembly, cell signaling and vesicular trafficking, intracellular survival/proteolysis, antioxidant defense, stress related proteins/protein folding, cell motility and cytoskeleton, nucleoside, nucleotide and nucleic acid metabolism, cell duplication/cell cycle, diverse cellular functions, DNA damage/DNA recombination/DNA repair (Table 3 and Fig. 3). In addition, some of these proteins are hypothetical proteins, which their functions in *Leishmania *remain to be elucidated. Most proteins identified in this study were assigned for the first time to a proteome map of *L. tropica*. The transcript level of some identified proteins was confirmed using real-time RT-PCR.

**Table 3:** Functional and Biological process categories of the identified proteins

**Spot No** a	**Protein name**			**Accession No ** b	**Induction factor (V/C) ** ^c^
**Carbohydrate metabolic process/Energy metabolism**					
*114*	*triosephosphate isomerase*			Q4QAP8	0.34
*131*	*hypothetical protein, conserved (ATP synthase B super family)* *			Q4Q079	0.47
*144*	*glycosomal malate dehydrogenase*			Q4QDF0	0.42
*255*	*cytosolic malate dehydrogenase*			Q4Q7X6	0.53
*273*	*NADP-dependent alcohol dehydrogenase, putative*			Q4QBD8	0.33
*274*	*NADP-dependent alcohol dehydrogenase, putative*			Q4QBD8	0.51
**Protein synthesis/assembly**					
*58*	*hypothetical protein, conserved (Alba superfamily)* *			Q4QGA9	0.37
*155*	*eukaryotic translation initiation factor 1A, putative*			Q4QF06	0.30
*161*	*hypothetical protein, conserved (Alba superfamily)* *			Q4Q2T7	0.18
*183*	*hypothetical protein, conserved (Alba superfamily)* *			Q4Q2T7	0.20
*428*	*elongation factor 1-alpha*			Q4QEI9	0.16
**Cell signaling and vesicular trafficking**					
*101*	*calmodulin-like protein*			Q4QF91	0.37
*119*	*vacuolar sorting-like protein*			Q4Q5H7	0.54
*154*	*Qc-SNARE protein, putative*			Q4QIG6	0.17
*324*	*ADP-ribosylation factor-like protein*			Q4Q756	0.32
*381*	*hypothetical protein, conserved (Enkurin superfamily)* *			Q4QAX0	0.04
*496*	*hypothetical protein, conserved (SNF-7-like protein)* *			Q4Q130	0.18
**Intracellular survival/Proteolysis**					
***120***	*hs1vu complex proteolytic subunit-like*			Q4Q116	2.03
***333***	*endoribonuclease L-PSP (pb5), putative*			Q4QBF5	2.38
***474***	*small ubiquitin protein, putative*			Q4QIC2	2.16
**Antioxidant defense/Oxidative stress defense**					
*206*	*Tryparedoxin*			E9ADX3	2.11
*457*	*peroxidoxin*			Q4QBH2	*DS*
**Stress related proteins/protein folding**					
*322*	*co-chaperone GrpE, putative*			Q4Q7N4	4.28
**Cell motility and cytoskeleton**					
*44*	*ADF/Cofilin*			E9ADQ2	0.21
*203*	*alpha tubulin*			Q4QGC5	0.39
**Nucleoside, nucleotide and nucleic acid metabolism**					
*353*	*adenylate kinase, putative*			Q4QC71	0.31
*377*	*dihydroorotate dehydrogenase*			Q4QEW7	0.21
**Cell duplication/Cell cycle**					
***70***	*hypothetical protein, conserved (Sfi1 spindle body protein)* *			Q4Q6E8	*DS*
**Table 3. (Continued).**					
**Spot No** a		**Protein name**	**Accession No ** b		**Induction factor (V/C) ** ^c^
**Diverse cellular functions**					
*359*	*hypothetical protein, conserved (P-loop_NTPase super family)* *			Q4QFN8	0.40
**DNA damage/DNA recomibination/DNA repair**					
*414*	*Structure-specific endonuclease subunit SLX1 homolog*			Q4Q9W0	0.48
**Hypotical protein/unknown function**					
*28*	*hypothetical protein*			Q4QFW1	0.51
*36*	*hypothetical protein*			Q4QFW1	*NDS*
*96*	*hypothetical protein, unknown function*			E9ACC9	8.38
*151*	*hypothetical protein, conserved*			Q4QED8	0.19
*327*	*hypothetical protein, unknown function*			Q4QEA4	*DS*
*438*	*hypothetical protein, conserved*			O97202	3.97
*449*	*hypothetical protein, conserved*			Q4QHN3	0.42
*450*	*hypothetical protein, conserved*			Q4QHN3	6.75

a The numbering corresponds to the 2-DE gel in Fig. 1./

b Accession number in Swiss-Prot./

g The induction factor calculated by dividing the percent volume of spots in gels corresponding to visceral leishmaniasis to the percent volume of spots in cutaneous leishmaniasis samples./* DS*: detected spots only in visceral leishmaniasis, *NDS*: not detected spots in visceraleishmaniasis./ Significantly up-regulated proteins in visceral leishmaniasis compared to cutaneous leishmaniasis./ Significantly down-regulated proteins in visceral leishmaniasis compared to cutaneous leishmaniasis.

* Hypothetical proteins with special domains

## Discussion

***Biological process of proteins identified***

Proteins with carbohydrate metabolism activity comprised the largest category (18%) found in this analysis. Six proteins in this group including triosephosphate isomerase (spot 114), hypothetical protein, conserved (ATP synthase B super family) (spot 131), two species of glycosomal and cytosolic malate dehydrogenase (spot 144 and 255) and two species of NADP-dependent alcohol dehydrogenase, putative (spot 273 and 274) showed down-regulation in the viscerotropic isolate (Table 3 and Fig. 3). 

The roles of these proteins have been implicated in glycolysis and the tricarboxylic acid cycle pathways that involved in the catabolism of glucose and energy production (20). Suppression of these proteins may suggest that the parasite might have reduced energy production and vital metabolism leading to decrease in multiplication of the parasite of immune system in viscera.

**Fig. 4:**
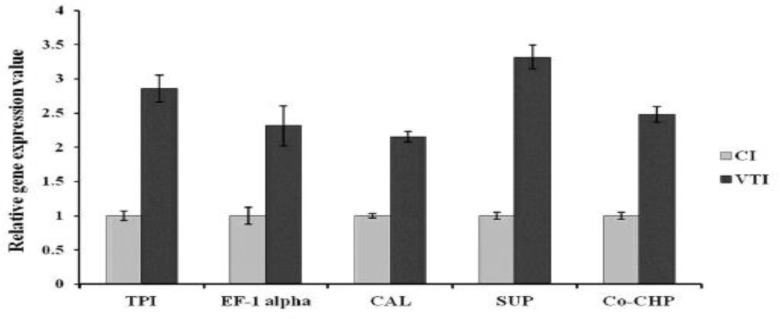
Relative gene expression pattern of target genes in cutaneous and visceral *L. tropica* isolates by real time RT-PCR. The expression of GAPDH was used to normalize the data. The values are the mean ± SD of three independent experiments (*P*<0.05).

Among this cluster TPI is one of the critical and important glycolytic enzymes, which was identified as a T- cell stimulatory protein and considered as a potential vaccine candidate against VL (21). Down-regulation of this protein in visceral form, might contribute to diminish elicitation of immune response and symptoms in disseminated *L. tropica*. Moreover, in order to address the relationship of protein expression levels with transcript levels, the mRNA expressions of TPI in cutaneous and viscerotropic *L. tropica* isolates were analyzed using real-time RT-PCR. Fig. 4 shows a significant up-regulation of TPI (2.855 Fold) in visceral isolate compared to cutaneous one (*P*<0.05) while it was down regulated at protein level (Table 3). The discrepancy between the expression of TPI at the level of protein and transcriptome indicating that post-transcriptional and post-translational regulation plays an important role in regulating of gene expression (22).

TPI: Triosephosphate isomerase, EF-1 alpha: Triosephosphate isomerase, CAL: Calmodulin-like protein, SUP: Small ubiquitin protein, Co-CHP: Co-chaperone GrpE

***Protein synthesis and assembly***

The second largest cluster of identified proteins (16%) consists of five down-regulated proteins (Table 3). These include three species of Alba super family (spots 58, 161 and 183), eukaryotic translation initiation factor 1A (TIF-1A) (spot 155) and Elongation factor-1alpha (EF-1alpha) (spot 428) which play a role in protein synthesis and assembly. Down-regulation of this cluster proposes that protein synthesis might be reduced in visceral form to reduce stimulation of immune response. Among this group, EF-1alpha deserves particular attention. It is a highly conserved GTP-binding protein involved in protein translation. In addition, it is an actin/microtubule-binding protein, which interacts with the cytoskeleton and recently in Leishmania, was identified as a virulence factor (23). This protein could diffuse into the cytosol of infected macrophages, where it is able to activate tyrosine phosphatase-1 leading to macrophage deactivation (24). Alternatively, it has been described as a potent antibody inducer involved in the humoral immune response during Mediterranean visceral leishmaniasis (25). Suppression of EF-1 alpha in visceral form could probably contribute to reduce elicitation of immune response and symptoms in viscerotropic form. Additionally, transcript level of EF-1 alpha was detected using real-time RT-PCR. Fig. 4 shows a significant up-regulation of EF-1 alpha (2.315 fold) in viscerotropic isolate in comparison with cutaneous one, which is in contrast with proteomics result, in which EF-1 alpha was down-regulated (Table 3). The inconsistency of results might be due to different post-translational regulation pathways such as changes in RNA stability (22).

***Cell signaling and vesicular trafficking***

Six of identified proteins were grouped in this cluster (spots 101, 119, 154, 324, 381 and 496) were down-regulated in VI (Table 3 and Fig. 3).One of the cell signaling proteins was calmodulin-like preotein (spot 101). Calmodulin is a kind of calcium binding protein, which expresses in all eukaryotic cells including members of the genus *Leishmania*; it participates in calcium signaling pathways that regulate multiple critical processes such as growth and proliferation (26). Moreover, it plays a vital role in virulence of *Leishmania* during invasion of macrophage (27). On the other hand, vacuolar sorting-like protein as a vesicular trafficking protein involved in sorting and delivering of vacuolar proteins to each intracellular compartment (28). In addition, this protein as a member of endosome sorting and authophagy pathways is essential for differentiation and virulence of *Leishmania *(29). Regarding down-regulation of this group, it is assumed that the parasite limited its central function in terms of cell signaling and vesicular trafficking to diminish induction of immune system. Furthermore, transcript level of CLP was up regulated about 2.156 fold in viscerotropic isolate compared to cutaneous (Fig. 4) which demonstrating of post-translational regulation. 

***Intracellular survival/Proteolysis***

We identified three proteins (spots 120, 333 and 474) were involved in intracellular survival/proteolysis category and were up regulated in VI (Table 3). This cluster consists of hs1vu complex proteolytic subunit-like (spot 120), endoribonuclease L-PSP (pb5), putative (spot 333) and small ubiquitin protein putative (spot 474). Of particular interest is ubiquitin, which plays a vital role in the protein degradation mechanism through the ubiquitin-proteasome pathway (UPP). This pathway is an important protein quality control mechanism for the selective proteolysis of oxidized and misfolded proteins, which prevents the cell from toxic accumulation of abnormal proteins (30). Over expression of ubiquitin might contribute to resistance to oxidative stress, heath shock and antimonial toxicity through degradation of oxidatively damaged proteins (31, 32). Therefore, our results suggest that the up-regulation of proteolysis proteins such as ubiquitin in viscerotropic form could promote degradation of oxidized proteins and thereby protect cell from oxidative stress and increased temperature the parasite encountered in viscera. 

Real-time RT-PCR result confirmed the expression pattern of small ubiquitin protein (SUP) in transcript level. As shown in Fig. 4, SUP was up-regulated (3.316 fold) in viscerotropic isolate compared to cutaneous one, which is in agreement with proteomics results (Table 3). 

***Antioxidant defense*** ***/Oxidative stress defense***

We identified two proteins involved in antioxidant defense or detoxification of reactive oxygen species (ROS). One protein, tryparedoxin (spot 206) upregulated up to 2.1 fold in visceral isolate and the other one (spot 484) identified as peroxidoxin was detected only in VI (Table 3). Tryparedoxins are special thiol disulfide oxidoreductases related to thioredoxins, which play a crucial role in hydroperoxide detoxification cascades of Kinetoplastida. They mediate electron transfer between trypanothione and a peroxiredoxin leading to reduce and detoxify hydroperoxides and possibly peroxynitrite (33). There is solid evidence that increased levels of tryparedoxins and possibly other components of the parasite hydroperoxide elimination apparatus in the cytosol could provide resistance to host-derived radicals leading to facilitate infection (34). Peroxidoxin as the other identified protein is a tryparedoxin peroxidase, which participates in antioxidant defense by decomposing ROS and reactive nitrogen species (RNS) with cooperation of tryparedoxins (35). Over-expression of peroxidoxin in *Leishmania* has enhanced resistance to oxidative stress induced by macrophage (36). Hence, enhancement in expression of tryparedoxin and peroxidoxin in visceral form might contribute to protection against macrophage released toxic oxidants and parasite survival in viscera.

***Stress related proteins/protein folding***

Co-chaperone GrpE (spot 322) was classified in the stress proteins category and was over-expressed up to 4.28 fold in visceral form (Table 3). In addition, RNA expression level of Co-chaperone GrpE was analyzed using Real-time RT-PCR. As shown in Fig. 4 Co-chaperone GrpE was up-regulated (2.479 fold) in viscerotropic isolate compared to cutaneous one, which is well consistent with proteomics result. 

Co chaperones are nonclient-binding partners of chaperones and are essential in the function of chaperones such as heat shock proteins (HSP). Co-chaperone GrpE are HSP70 co-chaperones regulating HSP70 action in protein folding (37). HSP 70 and its co-chaperones involved in different cellular processes such as assembly of newly synthesized proteins, refolding of misfolded proteins and regulation of the heat-shock and stress responses (38). Up-regulation of HSP70 protects cells from toxic effects of heat and oxidative stress due to preventing accumulation of misfolded proteins and cell death (39, 40). *L. tropica* increased co-chaperon expression to shield from increasing of temperature and oxidants, which encountered in dissemination to the viscera. 

***Cell motility and cytoskeleton***

This cluster consists of two down-regulated proteins in visceral isolate identified as ADF/Cofilin (spot 44) and alpha tubulin (spot 203) (Table 3 and Fig. 3). ADF/cofilin exists in all eukaryotic organisms and is involved in multiple actin-based cellular activities, such as cell motility and cytokinesis (41). *Leishmania *parasites express only one species of ADF/cofilin, which is essentially needed in flagellar assembly and motility (42).

Alpha tubulin is known as one of the members of distinct microtubule networks in *Leishmania*, which is implicated in locomotion, cell shape and division (43). Alpha tubulin has also been identified as a vaccine candidate antigen from a phage expression library using sera of VL patients (44). 

***Nucleoside, nucleotide and nucleic acid metabolism***

Two proteins recognized in this study belonged to nucleoside, nucleotide and nucleic acid metabolism. These include adenylate kinase putative (spot 353) and dihydroorotate dehydrogenase (spot 377), which both of them down regulated in VI (Table 3 and Fig. 3). There are different isoenzymic forms of adenylate kinase that catalyze the reversible transfer of the terminal phosphate group between ATP and AMP. This enzyme is involved in energy metabolism and nucleic acid synthesis, and is necessary for maintenance and cell growth (45). Earlier studies demonstrated that adenylate kinase may play a role in maintenance of ADP/ATP levels in *L. donovani* and it could be a potential target antigen for diagnosis or vaccination of leishmaniasis (46). Dihydroorotate dehydrogenas was another recognized protein classified in this category, which is the fourth enzyme in the pyrimidine biosynthetic pathway (47). This protein has been recognized as a potential virulence factors and drug targets in protozoan parasites (48). Down-regulation of aforementioned proteins in visceral form could conceivably reduce nucleotide acid metabolism.

***Cell duplication/Cell cycle***

In this group a hypothetical protein belonged to Sfi spindle body proteins (spot 70) was detected only in spleen (Table 3 and Fig. 3). In particular, Sfi1 is a centrin binding partner, which localizes to the half-bridge of the spindle pole body (SPB; the microtubule-organizing center) and has an essential function in SPB duplication and cell cycle (49). Moreover, depletion of this protein causes cell arrest with failure to form a mitotic spindle (50).

***Diverse cellular functions***

We identified a hypothetical protein associated with P-loop-NTPase superfamily (spot 359) which was down regulated in visceral form (Table 3 and Fig. 3). P-loop-NTPases represent a large protein super family and each subgroup member is involved in different cellular processes such as translation, signal transduction, metal insertion and protein transportation (51).

***DNA damage/DNA recombination/DNA repair***

A structure-specific endonulease subunit SLX homolog (spot 414) was classified in this group, which was down regulated in VI (Table 3 and Fig. 3). Structure-specific endonucleases are responsible for several kinds of DNA repair processes such as nucleotide excision repair and DNA inter strand crosslink repair (52). 

We recognized eight hypothetical proteins with unknown function. Additionally, homologous domains were not found through data analysis (Tables 2, 3 and Fig. 3). 

## Conclusion

*L. tropica* probably reduces the essential functions such as energy metabolisms, protein synthesis and cell signaling to lower its replication and induction of immune system contributing to establishment of infection in viscera. Moreover, down-regulation of some virulence factors such as elf1-alpha in visceral isolate could help parasite escape from immune system contributing to reduce host immune response and symptoms in viscerotropic form. Enhanced expression of co-chaperon, tryparedoxin and ubiquitin could assist parasite to shield from oxidants induced by immune system as well as higher temperature parasite exposed in viscera. 

Further investigations are required to explore precise function of these proteins in this scenario.

## Abbreviations

**ACL, **anthroponotic cutaneous leishmaniasis; **CI, **cutaneous isolate; **VTI (VI),** Viscerotropic(visceral) isolate**; 2 DE, **two-dimensional electrophoresis; **LC-MS,** liquid chromatography/mass Spectrometry; **Real-Time RT-PCR**, Real-time reverse transcription-polymerase chain reaction; **FBS,** fetal bovine serum; **NAGT**, N- acetylglucosamine-1- phosphate transferase; **PMSF**, phenylmethylsulfonyl fluoride; **DTT**, dithiothreitol; **TCA,** trichloroacetic acid; **BSA**, bovine serum albumin; **CHAPS,** (3-cholamidopropyl) dimethylammonio
